# Supplementary material for: Evolution shapes and conserves genomic signatures in viruses
Source: Commun Biol. 2024 Oct 30;7:1412. doi: 10.1038/s42003-024-07098-1 (PMC11526014; doi:10.1038/s42003-024-07098-1)
Supplement: Supplementary file 1 — Supplementary Information [file 42003_2024_7098_MOESM1_ESM.pdf]

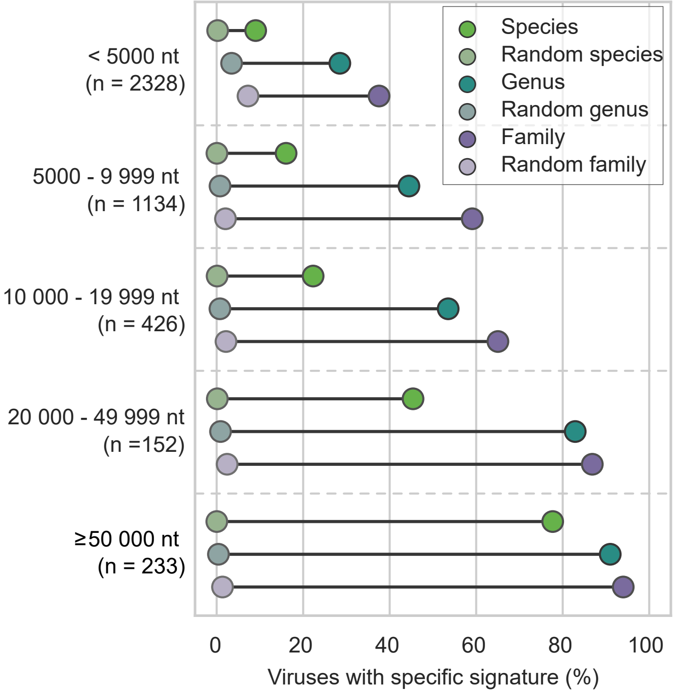

Species
Random species
Genus
Random genus
Family
Random family
< 5000 nt
(n = 2328)
5000 - 9 999 nt
(n = 1134)
10 000 - 19 999 nt
(n = 426)
20 000 - 49 999 nt
(n =152)
≥50 000 nt
(n = 233)
0
20
40
60
80
100
Viruses with specific signature (%)

Supplementary Figure 1. Statistical analysis of specificity. The percentage of viruses with species- (in green), genus- (in blue), and family-specific (in purple) compared to what would be expected by random chance (gray-green for species, gray-blue for genus, gray-purple for family). The viruses are subdivided by their respective sequence length. Our results demonstrate that the number of viruses with species-, genus-, and family-specific genomic signatures exceeds what would be expected by random chance ($p < 0.05$, Bonferroni adjusted).

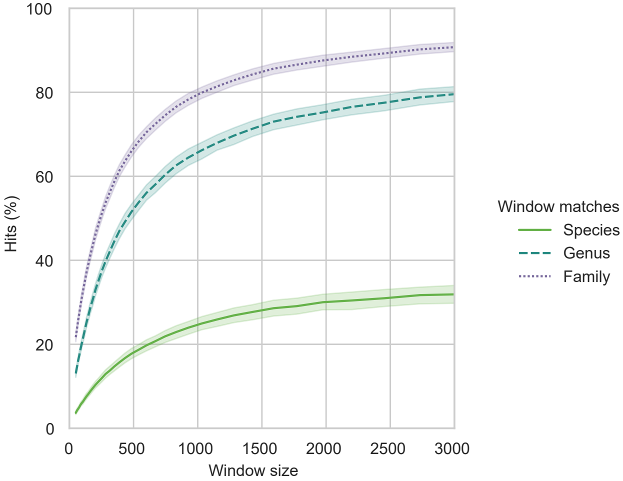

Hits (%)
0
20
40
60
80
100
0
500
1000
1500
2000
2500
3000
Window size
Window matches
Species
Genus
Family

Supplementary Figure 2. Detection of genomic signatures for different sequence lengths. To estimate the average sequence length needed for classification, subsequences were randomly selected for all viruses with genomes larger than 10,000 nt. The average percentage of sequences that match the correct species, genus, or family in relation to the window size is colour coded and plotted corresponding to sequence length. The shaded area around each line corresponds to a 95% confidence interval.

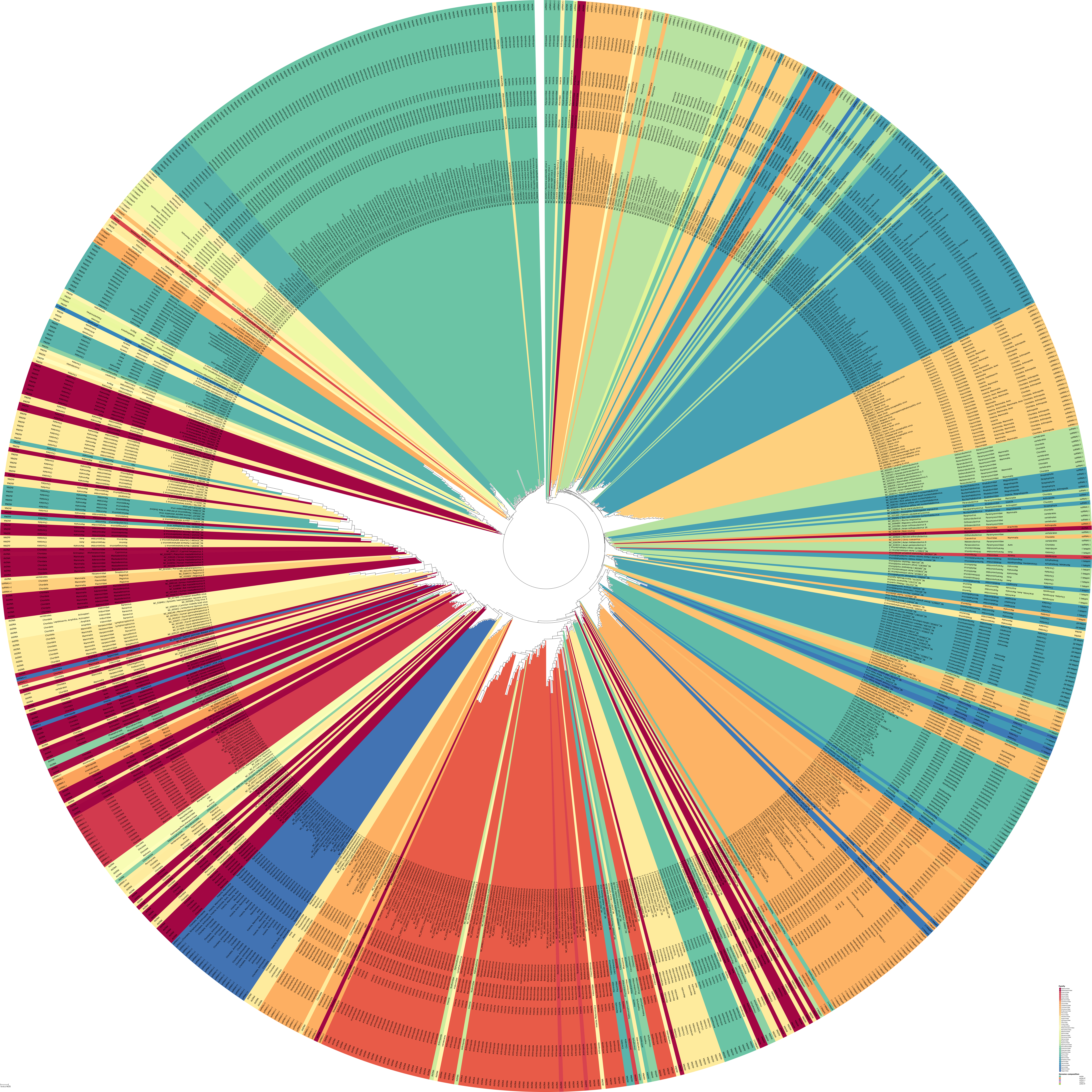

0.0117635
Family
Adenoviridae
Alloherpesviridae
Arteriviridae
Artoviridae
Ascoviridae
Asfarviridae
Baculoviridae
Caulimoviridae
Chuviridae
Closteroviridae
Coronaviridae
Dicistroviridae
Endornaviridae
Filoviridae
Flaviviridae
Herpesviridae
Hypoviridae
Hytrosaviridae
Iflaviridae
Iridoviridae
Lavidaviridae
Malacoherpesviridae
Marseilleviridae
Mesoniviridae
Mimiviridae
Mononiviridae
Mymonaviridae
Nairoviridae
Nudiviridae
Nyamiviridae
Paramyxoviridae
Phycodnaviridae
Pneumoviridae
Polycipiviridae
Polydnaviridae
Potyviridae
Poxviridae
Retroviridae
Rhabdoviridae
Roniviridae
Secoviridae
Solinviviridae
Sunviridae
Tobaniviridae
Togaviridae
Genome composition
dsDNA
dsDNA-RT
ssRNA(+)
ssRNA(-)
ssRNA-RT

Supplementary Figure 3. Clustering of viruses based on their genomic signatures. The complete unrooted neighbour-joining tree that is presented in Fig. 5, with additional data for each viral species. The tree includes all viruses with genomes larger than 10,000 nt and the distances between taxa correspond to similarities in genomic signature (the shorter the distance, the more similar genomic signatures).

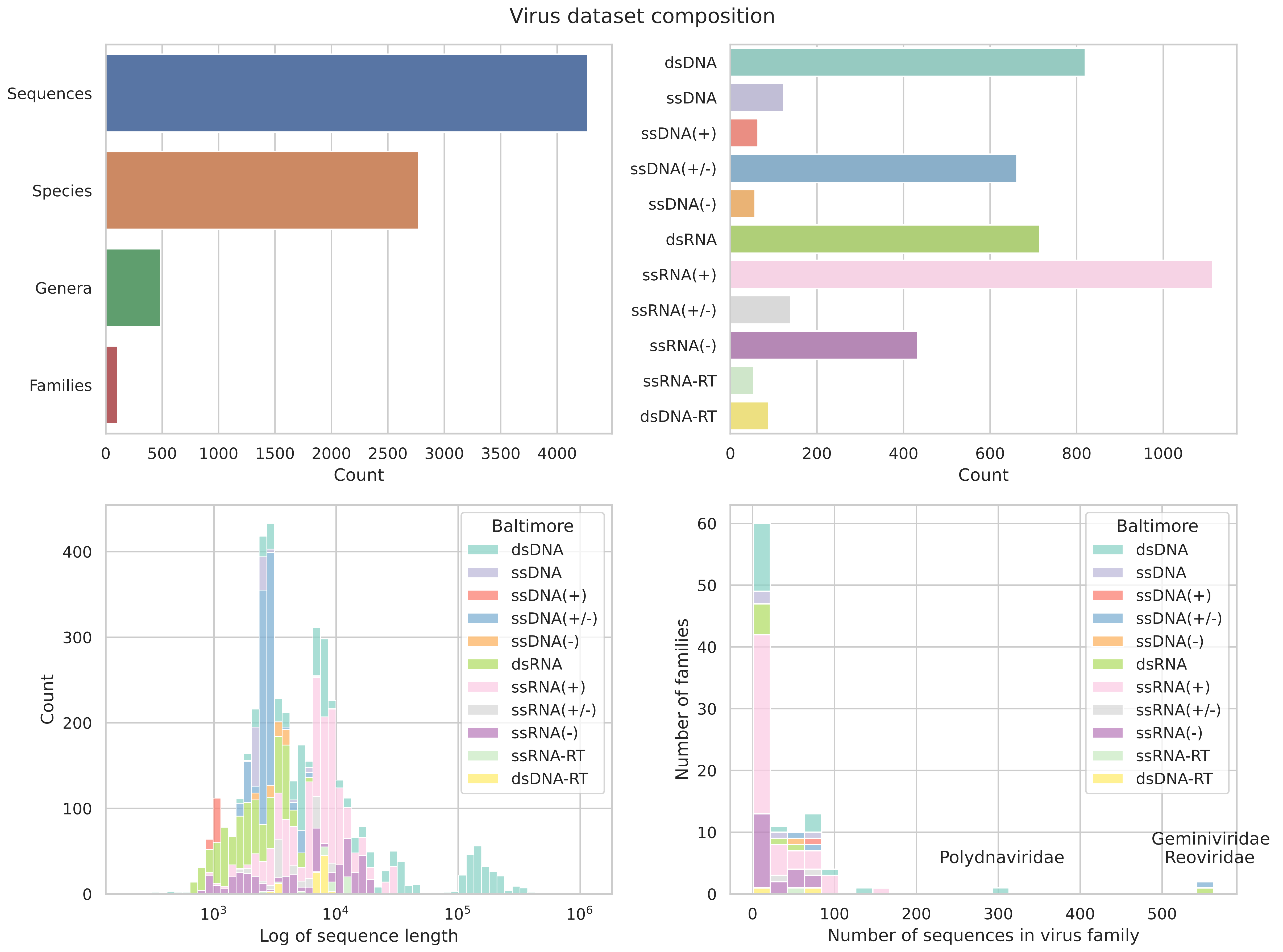
Virus dataset composition
Sequences
Species
Genera
Families
Count
dsDNA
ssDNA
ssDNA(+)
ssDNA(+/-)
ssDNA(-)
dsRNA
ssRNA(+)
ssRNA(+/-)
ssRNA(-)
ssRNA-RT
dsDNA-RT
Count
Baltimore
Count
Log of sequence length
Number of families
Number of sequences in virus family
Polydnaviridae
Geminiviridae
Reoviridae

Supplementary Figure 4. Composition of the viral genome dataset. Illustration of the number of sequences, species, genera, and families are shown. In addition, the number of sequences within each Baltimore class and the respective sequence length of those viruses are illustrated. Lastly, the number of sequences per family is included, where a few families are represented by significantly more sequences than other.

group	rank	adjusted p value
dsDNA	species	2.18E-16
dsDNA	genus	6.03E-17
dsDNA	family	9.82E-13
dsDNA-RT	species	0
dsDNA-RT	genus	6.23E-10
dsDNA-RT	family	1.67E-10
dsRNA	species	1.22E-11
dsRNA	genus	3.87E-11
dsRNA	family	1.05E-11
ssDNA	species	0
ssDNA	genus	1.84E-09
ssDNA	family	1.75E-08
ssDNA(-)	species	0
ssDNA(-)	genus	5.78E-05
ssDNA(-)	family	0.000176
ssDNA(+)	species	1
ssDNA(+)	genus	0.627981
ssDNA(+)	family	0.045695
ssDNA(+/-)	species	7.62E-08
ssDNA(+/-)	genus	5.51E-08
ssDNA(+/-)	family	3.08E-07
ssRNA(-)	species	1.01E-12
ssRNA(-)	genus	1.24E-12
ssRNA(-)	family	2.07E-13
ssRNA(+)	species	2.73E-20
ssRNA(+)	genus	9.71E-17
ssRNA(+)	family	1.65E-14
ssRNA(+/-)	species	1.59E-06
ssRNA(+/-)	genus	1.52E-12
ssRNA(+/-)	family	4.31E-12
ssRNA-RT	species	0
ssRNA-RT	genus	5.93E-14
ssRNA-RT	family	7.06E-09

Supplementary Table 1. Adjusted p values from the test of number of viruses with specific signatures per Baltimore group and taxonomic rank.

Similar to host in rank	aid	Virus species	Virus genus	Virus family	Host kingdom(s)	Host phylum(s)
species	NC_001362	Murine leukemia virus	Gammaretrovirus	Retroviridae	Metazoa	Chordata
species	NC_003397	Bean common mosaic virus	Potyvirus	Potyviridae	Viridiplantae	Streptophyta
species	NC_008393	Pepper severe mosaic virus	Potyvirus	Potyviridae	Viridiplantae	Streptophyta
species	NC_011918	Pepper veinal mottle virus	Potyvirus	Potyviridae	Viridiplantae	Streptophyta
species	NC_024451	Invertebrate iridescent virus 31	Iridovirus	Iridoviridae	Metazoa	Arthropoda
species	NC_030446	Tibetan frog hepatitis B virus	Herpetohepadnavirus	Hepadnaviridae	Metazoa	Chordata
genus	NC_004782	Mirafiori lettuce big-vein ophiovirus	Ophiovirus	Aspiviridae	Viridiplantae	Streptophyta
genus	NC_025388	Asian prunus virus 1	Foveavirus	Betaflexiviridae	Viridiplantae	Streptophyta
genus	NC_028868	Asian prunus virus 2	Foveavirus	Betaflexiviridae	Viridiplantae	Streptophyta
family	NC_001641	Saccharomyces cerevisiae virus LBCLa	Totivirus	Totiviridae	Fungi	Ascomycota
family	NC_003084	Culex nigripalpus nucleopolyhedrovirus	Deltabaculovirus	Baculoviridae	Metazoa	Arthropoda
family	NC_003649	Pelargonium zonate spot virus	Anulavirus	Bromoviridae	Viridiplantae	Streptophyta
family	NC_003738	Red clover mottle virus	Comovirus	Secoviridae	Viridiplantae	Streptophyta
family	NC_004573	Peru tomato mosaic virus	Potyvirus	Potyviridae	Viridiplantae	Streptophyta
family	NC_008300	Melon yellow spot orthotospovirus	Orthotospovirus	Tospoviridae	Viridiplantae	Streptophyta
family	NC_015395	Hardenbergia virus A	Divavirus	Betaflexiviridae	Viridiplantae	Streptophyta
family	NC_017824	Tomato necrotic stunt virus	Potyvirus	Potyviridae	Viridiplantae	Streptophyta
family	NC_018071	Bean necrotic mosaic orthotospovirus	Orthotospovirus	Tospoviridae	Viridiplantae	Streptophyta
family	NC_043537	African eggplant mosaic virus	Potyvirus	Potyviridae	Viridiplantae	Streptophyta
order	NC_003003	Broad bean wilt virus 2	Fabavirus	Secoviridae	Viridiplantae	Streptophyta
order	NC_003645	Milk vetch dwarf virus	Nanovirus	Nanoviridae	Metazoa, Viridiplantae	Arthropoda, Streptophyta
order	NC_003687	Powassan virus	Flavivirus	Flaviviridae	Metazoa	Chordata, Arthropoda
order	NC_004778	Choristoneura fumiferana multiple nucleopolyhedrovirus	Alphabaculovirus	Baculoviridae	Metazoa	Arthropoda
order	NC_005906	Neodiprion lecontei nucleopolyhedrovirus	Gammabaculovirus	Baculoviridae	Metazoa	Arthropoda
order	NC_006653	Cotesia congregata bracovirus	Bracovirus	Polydnaviridae	Metazoa	Arthropoda
order	NC_006961	Pleurotus ostreatus virus 1	Betapartitivirus	Partitiviridae	Fungi	Basidiomycota
order	NC_007985	Campoletis sonorensis ichnovirus	Ichnovirus	Polydnaviridae	Metazoa	Arthropoda
order	NC_008035	Antheraea pernyi nucleopolyhedrovirus	Alphabaculovirus	Baculoviridae	Metazoa	Arthropoda
order	NC_008837	Glypta fumiferanae ichnovirus	Ichnovirus	Polydnaviridae	Metazoa	Arthropoda
order	NC_008947	Hyposoter fugitivus ichnovirus	Ichnovirus	Polydnaviridae	Metazoa	Arthropoda
order	NC_009246	Rice gall dwarf virus	Phytoreovirus	Reoviridae	Metazoa, Viridiplantae	Arthropoda, Streptophyta
order	NC_011335	Diadromus pulchellus toursvirus	Toursvirus	Ascoviridae	Metazoa	Arthropoda
order	NC_011588	Oryctes rhinoceros nudivirus	Alphanudivirus	Nudiviridae	Metazoa	Arthropoda
order	NC_017970	Sweet potato virus 2	Potyvirus	Potyviridae	Viridiplantae	Streptophyta
order	NC_018093	Sweet potato virus G	Potyvirus	Potyviridae	Viridiplantae	Streptophyta
order	NC_021924	Choristoneura rosaceana nucleopolyhedrovirus	Alphabaculovirus	Baculoviridae	Metazoa	Arthropoda
order	NC_023016	Lamium mild mosaic virus	Fabavirus	Secoviridae	Viridiplantae	Streptophyta
order	NC_023177	Choristoneura murinana nucleopolyhedrovirus	Alphabaculovirus	Baculoviridae	Metazoa	Arthropoda
order	NC_024684	Lizard atadenovirus A	Atadenovirus	Adenoviridae	Metazoa	Chordata
order	NC_025481	Lilac leaf chlorosis virus	Ilarvirus	Bromoviridae	Viridiplantae	Streptophyta
order	NC_033780	Mythimna unipuncta granulovirus B	Betabaculovirus	Baculoviridae	Metazoa	Arthropoda
order	NC_038697	Scheffersomyces segobiensis virus L	Totivirus	Totiviridae	Fungi	Ascomycota
order	NC_038916	Chondrostereum purpureum cryptic virus 1	Alphapartitivirus	Partitiviridae	Fungi	Basidiomycota
order	NC_043270	Chelonus inanitus bracovirus	Bracovirus	Polydnaviridae	Metazoa	Arthropoda
order	NC_043324	Diolcogaster facetosa bracovirus	Bracovirus	Polydnaviridae	Metazoa	Arthropoda

Supplementary Table 2. The viruses that are significantly similar to host from the same species, genus, family, or order.
