# Supplementary material for: Evolution shapes and conserves genomic signatures in viruses
Source: Commun Biol. 2024 Oct 30;7:1412. doi: 10.1038/s42003-024-07098-1 (PMC11526014; doi:10.1038/s42003-024-07098-1)
Supplement: Supplementary file 2 — Description of Additional Supplementary Materials [file 42003_2024_7098_MOESM2_ESM.pdf]

Description of Additional Supplementary Files

File name: Supplementary Table 3

Description: Virus sequence dataset

File name: Supplementary Table 4

Description: Host sequence dataset
