# Supplementary material for: Evolution shapes and conserves genomic signatures in viruses
Source: Commun Biol. 2024 Oct 30;7:1412. doi: 10.1038/s42003-024-07098-1 (PMC11526014; doi:10.1038/s42003-024-07098-1)
Supplement: Supplementary file 5 — Reporting Summary [file 42003_2024_7098_MOESM5_ESM.pdf]

nature portfolio

Corresponding author(s): DAPR, COMMSBIO-23-5030-T

Last updated by author(s): Jan 10, 2024

Reporting Summary

Nature Portfolio wishes to improve the reproducibility of the work that we publish. This form provides structure for consistency and transparency in reporting. For further information on Nature Portfolio policies, see our Editorial Policies and the Editorial Policy Checklist.

Statistics

For all statistical analyses, confirm that the following items are present in the figure legend, table legend, main text, or Methods section.

n/a	Confirmed	
☐	☒	The exact sample size (*n*) for each experimental group/condition, given as a discrete number and unit of measurement
☐	☒	A statement on whether measurements were taken from distinct samples or whether the same sample was measured repeatedly
☐	☒	The statistical test(s) used AND whether they are one- or two-sided<br>*Only common tests should be described solely by name; describe more complex techniques in the Methods section.*
☐	☒	A description of all covariates tested
☐	☒	A description of any assumptions or corrections, such as tests of normality and adjustment for multiple comparisons
☐	☒	A full description of the statistical parameters including central tendency (e.g. means) or other basic estimates (e.g. regression coefficient) AND variation (e.g. standard deviation) or associated estimates of uncertainty (e.g. confidence intervals)
☐	☒	For null hypothesis testing, the test statistic (e.g. *F*, *t*, *r*) with confidence intervals, effect sizes, degrees of freedom and *P* value noted<br>*Give P values as exact values whenever suitable.*
☐	☒	For Bayesian analysis, information on the choice of priors and Markov chain Monte Carlo settings
☐	☒	For hierarchical and complex designs, identification of the appropriate level for tests and full reporting of outcomes
☒	☐	Estimates of effect sizes (e.g. Cohen's *d*, Pearson's *r*), indicating how they were calculated

Our web collection on statistics for biologists contains articles on many of the points above.

Software and code

Policy information about availability of computer code

Data collection	The dataset of eukaryotic viruses was based on the ICTV Master Species List (2018b.v2). We identified viral hosts using VirusHostDB
Data analysis	All custom code required to run our analysis is available at https://gitlab.com/genomic-signatures/genomic-signatures-in-viruses.

For manuscripts utilizing custom algorithms or software that are central to the research but not yet described in published literature, software must be made available to editors and reviewers. We strongly encourage code deposition in a community repository (e.g. GitHub). See the Nature Portfolio guidelines for submitting code & software for further information.

Data

Policy information about availability of data

All manuscripts must include a data availability statement. This statement should provide the following information, where applicable:

- Accession codes, unique identifiers, or web links for publicly available datasets
- A description of any restrictions on data availability
- For clinical datasets or third party data, please ensure that the statement adheres to our policy

Accession id, name, taxonomy, and sequence length for each genomic sequence analyzed here are available in Supplementary Data 3 and 4.

Research involving human participants, their data, or biological material

Policy information about studies with human participants or human data. See also policy information about sex, gender (identity/presentation), and sexual orientation and race, ethnicity and racism.

Reporting on sex and gender	N/A
Reporting on race, ethnicity, or other socially relevant groupings	N/A
Population characteristics	N/A
Recruitment	N/A
Ethics oversight	N/A

Note that full information on the approval of the study protocol must also be provided in the manuscript.

Field-specific reporting

Please select the one below that is the best fit for your research. If you are not sure, read the appropriate sections before making your selection.

☐ Life sciences ☐ Behavioural & social sciences ☒ Ecological, evolutionary & environmental sciences

For a reference copy of the document with all sections, see nature.com/documents/nr-reporting-summary-flat.pdf

Ecological, evolutionary & environmental sciences study design

All studies must disclose on these points even when the disclosure is negative.

Study description	We investigate the presence and specificity of genomic signatures in eukaryotic viruses, aiming to illuminate dependencies and selective pressures in viral genome evolution
Research sample	We analyzed the genomes of 2,768 eukaryotic viral species from 105 viral families, and 2,399 eukaryotic genomes: 1,267 fungi, 873 animals and insects, 234 plants, and 25 protists
Sampling strategy	We included all complete genomes from the dataset of eukaryotic viruses in the ICTV Master Species List (2018b.v2), and coding sequences of each host from NCBI identified using VirusHostDBavailable at NCBI
Data collection	All sequences were downloaded from NCBI
Timing and spatial scale	The sequences were downloaded 2018
Data exclusions	Incomplete and partial sequences were excluded
Reproducibility	To obtain a comprehensive understanding, we employed a sampling methodology, which was reiterated 1,000 times to gauge the significance of our findings. This approach randomly paired each query to a profile. Then, we computed the matches to the correct species, genus, and family for each virus, providing insight into the anticipated number of random matches, not considering the genomic signatures. We also conducted a Bonferroni corrected two-tailed t-test to compare the quantities of specific signatures in both the random analysis and the analyses based on genomic signatures. We considered adjusted p-values less than 0.05 as statistically significant.
Randomization	Viral sequences were ordered in groups according to species, genus, families, and Baltimore classification
Blinding	Blinding was not relevant for the study

Did the study involve field work? ☐ Yes ☒ No

Reporting for specific materials, systems and methods

We require information from authors about some types of materials, experimental systems and methods used in many studies. Here, indicate whether each material, system or method listed is relevant to your study. If you are not sure if a list item applies to your research, read the appropriate section before selecting a response.

Materials & experimental systems

n/a	Involved in the study
☒	☐ Antibodies
☒	☐ Eukaryotic cell lines
☒	☐ Palaeontology and archaeology
☒	☐ Animals and other organisms
☒	☐ Clinical data
☒	☐ Dual use research of concern
☒	☐ Plants

Methods

n/a	Involved in the study
☒	☐ ChIP-seq
☒	☐ Flow cytometry
☒	☐ MRI-based neuroimaging

Plants

Seed stocks	N/A
Novel plant genotypes	N/A
Authentication	N/A
